# A Novel Reconstruction Technique to Reduce Stair-Step Artifacts in Sequential Mode Coronary CT Angiography

**DOI:** 10.1097/RLI.0000000000001066

**Published:** 2024-01-30

**Authors:** Lukas Jakob Moser, Victor Mergen, Thomas Allmendinger, Robert Manka, Matthias Eberhard, Hatem Alkadhi

**Affiliations:** From the Diagnostic and Interventional Radiology, University Hospital Zurich, University of Zurich, Zurich, Switzerland (L.J.M., V.M., R.M., M.E., H.A.); Siemens Healthineers AG, Forchheim, Germany (T.A.); and Department of Radiology, Spital Interlaken, Spitäler fmi AG, Unterseen, Switzerland (M.E.).

**Keywords:** computed tomography, coronary angiography, sequential, step and shoot, stair-step, artifact, ZeeFree, reconstruction

## Abstract

**Purpose:**

Prospective electrocardiography-triggering is one of the most commonly used cardiac computed tomography (CT) scan modes but can be susceptible to stair-step artifacts in the transition areas of an acquisition over multiple cardiac cycles. We evaluated a novel reconstruction algorithm to reduce the occurrence and severity of such artifacts in sequential coronary CT angiography.

**Materials and Methods:**

In this institutional review board–approved, retrospective study, 50 consecutive patients (16 females; mean age, 58.9 ± 15.2) were included who underwent coronary CT angiography on a dual-source photon-counting detector CT in the sequential ultra-high-resolution mode with a detector collimation of 120 × 0.2 mm. Each scan was reconstructed without (hereafter called standard reconstruction) and with the novel ZeeFree reconstruction algorithm, which aims to minimize stair-step artifacts. The presence and extent of stair-step artifacts were rated by 2 independent, blinded readers on a 4-point discrete visual scale. The relationship between the occurrences of artifacts was correlated with the average and variability of heart rate and with patient characteristics.

**Results:**

A total of 504 coronary segments were included into the analyses. In standard reconstructions, reader 1 reported stair-step artifacts in 40/504 (7.9%) segments, from which 12/504 led to nondiagnostic image quality (2.4% of all segments). Reader 2 reported 56/504 (11.1%) stair-step artifacts, from which 11/504 lead to nondiagnostic image quality (2.2% of all segments). With the ZeeFree algorithm, 9/12 (75%) and 8/11 (73%) of the nondiagnostic segments improved to a diagnostic quality for readers 1 and 2, respectively. The ZeeFree reconstruction algorithm significantly reduced the frequency and extent of stair-step artifacts compared with standard reconstructions for both readers (*P* < 0.001, each). Heart rate variability and body mass index were significantly related to the occurrence of stair-step artifacts (*P* < 0.05).

**Conclusions:**

Our study demonstrates the feasibility and effectiveness of a novel reconstruction algorithm leading to a significant reduction of stair-step artifacts and, hence, a reduction of coronary segments with a nondiagnostic image quality in sequential ultra-high-resolution coronary photon-counting detector CT angiography.

Various scan acquisition techniques exist for coronary computed tomography angiography (CCTA) including retrospective electrocardiography (ECG)-gated spiral acquisitions, prospective ECG-triggered sequential acquisitions, and prospective ECG-triggered high-pitch spiral acquisitions.^1^ Each of these acquisition modes has specific characteristics and is associated with certain advantages and disadvantages.

Prospective ECG-triggered sequential scanning is one of the most commonly performed acquisition modes and is characterized by providing diagnostic image quality at a reasonably low radiation dose.^2,3^ One downside of the sequential acquisition mode is the potential occurrence of stair-step artifacts with a reported prevalence ranging from 18%^4^ to 78%.^5^ Potential reasons for the occurrence of stair-step artifacts include elevated heart rates (HRs) during data acquisition, HR variability, high body mass index (BMI) and body weight, and insufficient patient breath-hold.^6–9^

When considering CCTA as a test on an intent-to-diagnose basis, each segment with nondiagnostic image quality must be considered positive for coronary stenosis, since relevant coronary artery disease (CAD) cannot be safely ruled out.^10^ This not only limits the diagnostic yield of the modality, but also weakens the performance characteristics by lowering of the specificity and positive predictive value.

Recently, a new algorithm has been developed, which performs a nonrigid registration between the borders of 2 adjacent subvolumes acquired in the sequential technique, with the aim of minimizing the occurrence of stair-step artifacts. The purpose of this study was to evaluate this novel reconstruction algorithm to reduce the occurrence and severity of such artifacts in sequential CCTA.

## MATERIALS AND METHODS

### Patient Population

This retrospective study was performed at a tertiary hospital and had institutional review board and ethics committee agreement. All patients provided general consent for further use of their data for anonymized research.

**FIGURE 1:**
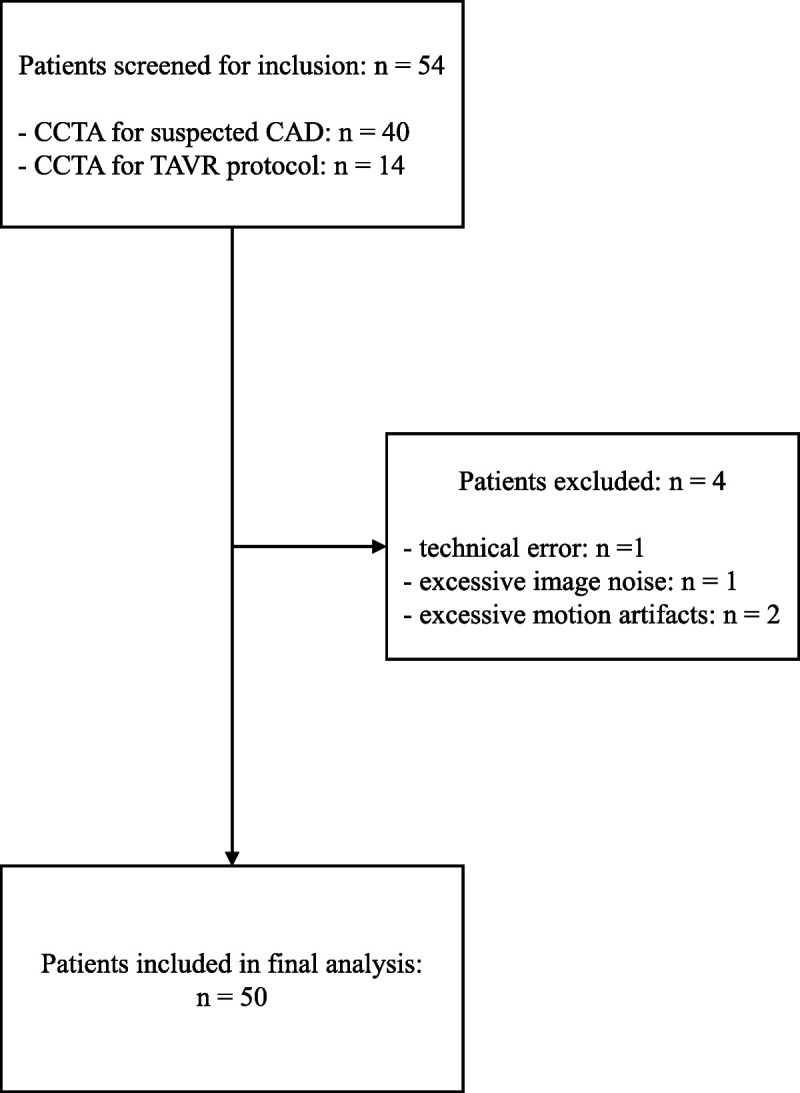
Patient flowchart.

A total number of 54 consecutive patients who were referred for CCTA and who were scanned in the sequential ECG-triggered mode between August and October 2023 were screened for study inclusion. In 40 patients, the indication was suspected CAD or progression of known CAD. In the remaining 14 patients, CCTA was performed as part of a transcatheter aortic valve replacement planning protocol. One patient was excluded because of a technical error resulting in a fixed acquisition in early systole. One patient was excluded for excessive image noise resulting in nondiagnostic images (BMI, 37 kg/m^2^). Two patients were excluded because of excessive motion artifacts. Thus, 50 patients (16 female, 34 male; mean age, 58.9 ± 15.2 years) were included in the final analysis (Fig. 1, Table 1).

**TABLE 1:** Baseline Characteristics

Characteristic	Value (n = 50)
Sex	
F	16 (32%)
M	34 (68%)
Age, y	58.9 ± 15.2 (range, 22–86)
Body weight, kg	74.7 ± 14.0 (range, 42–108)
Body mass index, kg/m^2^	24.9 ± 4.1 (range, 16.8–38.9)
Heart rate during acquisition, bpm	73.0 ± 13.2 (range, 51.8–101.3)
Heart rate variability (SD), bpm*^,†^	2.9 (2.0–4.1)
Heart rate variability (range), bpm^†,‡^	8 (5–11)
Medical history	
Arterial hypertension	25 (50%)
Diabetes	4 (8%)
Dyslipidemia	18 (36%)
Smoking history	17 (34%)

Note: Unless otherwise indicated, data are mean ± standard deviation or number of patients with percentages in parentheses. n = number of patients, bpm = beats per minute.

*Heart rate variability calculated as standard deviation from mean heart rate.

†Data are median; data in parentheses are interquartile range.

‡Heart rate variability calculated as range of heart rates during data acquisition.

### CT Scan and Image Reconstruction Parameters

In this study, we collected consecutive ultra-high-resolution (Quantum HD Cardiac; Siemens) CCTA scans on a clinical dual-source PCD-CT (NEAOTOM Alpha, Software Version VB10; Siemens Healthcare GmbH, Erlangen, Germany). Scans were acquired in a sequential, prospectively ECG-triggered mode using a slip ring data rate limited narrow detector collimation of 120 × 0.2 mm. Tube voltage was 120 kV, and image quality level was set to 64 using automated tube current-modulation (CARE Dose4D). The temporal resolution of data acquisition was 66 milliseconds.^11^ Electrocardiography-padding was applied,^12^ which results in the acquisition of multiple cardiac phases depending on the individual HR. The median CTDI_vol_ was 31.2 mGy (interquartile range, 23.9–40.1 mGy).

Coronary computed tomography angiography was acquired with a triphasic contrast media protocol applying 60–100 mL contrast media (iopromide, Ultravist 370 mg I/mL; Bayer Healthcare, Berlin, Germany) at an injection rate between 3.2 mL/s and 6.0 mL/s, depending on the patients' BMI. All scans were initiated after a threshold of 140 Hounsfield units (HU) at 90 kV was reached in the ascending aorta using the bolus-tracking technique. Patients were premedicated with sublingual nitroglycerin (2.5 mg isosorbide dinitrate), unless contraindicated. No β-blocker medication for HR control was administered.

Images were reconstructed with a field of view of 200 × 200 mm, a matrix of 512 × 512 pixels, and using a sharp vascular kernel (Bv60) with quantum iterative reconstruction at strength level 4.^13^ Slice thickness was 0.2 mm, increment was 0.2 mm, and the diastolic and systolic %-phases showing least motion artifacts derived from an automatic algorithm^14^ were chosen. Of these 2 reconstruction phases, the single best phase was then selected manually by an unblinded member of the study team (L.J.M., first-year radiology resident). Each scan was reconstructed in the scanner's standard reconstruction mode using an interpolation step, and with the novel ZeeFree reconstruction algorithm.

### The ZeeFree Algorithm

Obtaining whole heart data in sequential ECG-triggered cardiac CT requires the acquisition of individual subvolumes limited to the detector coverage of the selected scan mode over multiple cardiac cycles. These acquisitions are performed with a data overlap of approximately 10%. The application of the ZeeFree misalignment correction algorithm to this kind of data can be broken down into 3 separate steps:

As initial step, a weighted filtered back projection image reconstruction is performed separately for each contributing cardiac cycle with partially fixed parameters to ensure independence in the performance from user specific settings (eg, reconstruction kernel, matrix size). These images have a z-coverage of a single full detector collimation and are reconstructed with enabled slice saturation, a process that allows contributions to the back projection process outside of the covered detector with a very small weight, to avoid data gaps toward the outer field of view in a cone-beam geometry.^15^

**FIGURE 2:**
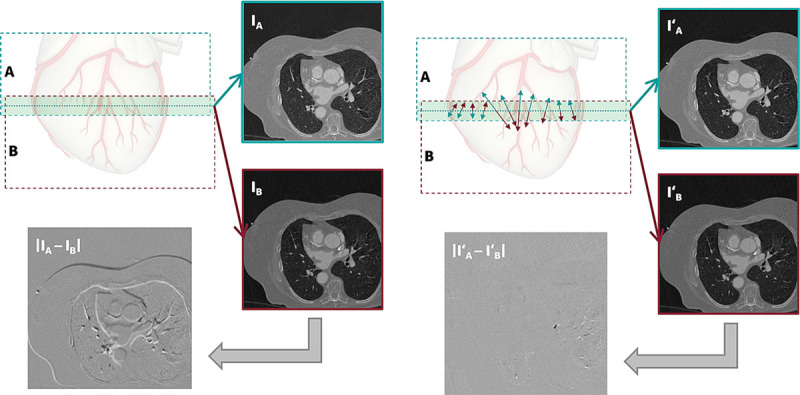
Illustration of the registration principle of the ZeeFree algorithm.

As second step, a displacement 3D vector field for each individual subvolume stack transition is derived. In each transition, the center of the overlap region defines a single image position, which allows to select a single image pair from both contributing subvolumes (Fig. 2). These 2 images I_A_ and I_B_ are subsequently subjected to a demon type registration algorithm,^16,17^ based on a joint 3D vector field model with opposing sign using the full volumetric data accessible in each subvolume in all 3 spatial directions and minimizing the root-mean-squared of the difference of the 2 images (see Fig. 2).

In the final step, all individually derived 3D vector fields are transition distance-weighted, interpolated, and resampled to a single whole heart field. This distance-weighted interpolation step contains a Gaussian smoothing operation, which can be interpreted as a relaxation of the “tension” in the vector field over the distance from the transition. As a final step, the full volume 3D vector field is applied to a dedicated reconstruction performed with all user-defined reconstruction parameters (eg, kernel, slice thickness, field of view, matrix size, iterative reconstruction).

The ZeeFree algorithm is an independent development with some of the basic principles taken from Lebedev et al,^18^ which also provides a more detailed mathematical discussion on the solution of this type of problem.

### Subjective and Objective Image Readout

Two independent, blinded readers (H.A. with 15 years of experience in cardiovascular imaging and M.E. with 10 years of experience in cardiovascular imaging) performed the subjective readout using multiplanar reformations. The evaluation was performed for the segments of the coronary artery tree as defined by the American Heart Association.^19^ Segments 1–4 correspond to the right coronary artery (RCA), 5 to the left main artery, 6–10 to the left anterior descending artery and diagonal branches, and 11–15 to the left circumflex artery and marginal branches. Segments with a diameter below 1.5 mm were not included in the evaluation.

Each segment was scored on a 4-point visual grading scale: a score of 1 indicated no stair-step artifacts, score 2 indicated small stair-step artifacts of less than 25% of the vessel diameter, score 3 indicated moderate star-step artifacts of less than the vessel diameter, and a score of 4 denoted a stair-step artifact with considerable discontinuity of the vessel. Scores of 1–3 were considered diagnostic, whereas a score of 4 was considered to indicate a nondiagnostic image quality.

Image noise, signal-to-noise ratio (SNR), and contrast-to-noise ratio (CNR) were calculated in both CCTA datasets as follows: regions of interest were placed in the ascending aorta at the level of the origin of the left main artery and in the epicardial adipose tissue (EAT) surrounding the proximal RCA. Average attenuation and standard deviation (SD) of attenuation were recorded. Image noise was defined as SD of attenuation in the ascending aorta.

SNR = HU_Aorta_/SD_Aorta_

CNR = (HU_Aorta_ − HU_EAT_)/SD_Aorta_

HR variability was calculated both as the SD of mean HR (HRV_SD_) during data acquisition^20,21^ and as the range of the HR during data acquisition (HRV_range_ = HR_maximum_ − HR_minimum_).^7^

## STATISTICAL ANALYSIS

All analyses were performed in R (version 4.3.1, The R Foundation). Normality of distribution was determined by Shapiro-Wilk testing. Quantitative variables are expressed as mean ± standard deviation or median and interquartile range (IQR), as applicable. Qualitative variables are reported as counts or percentages. Interrater agreement was calculated as Krippendorff α coefficients (0, no agreement; 1, perfect agreement). Nominal variables were compared by Fisher exact test or McNemar test, as appropriate. Wilcoxon tests were used to compare ordinal and continuous variables. *T* tests were applied to compare normally distributed continuous variables. Statistical significance was assumed at a 2-tailed *P* value <0.05.

## RESULTS

In the 50 patients, a total of 504 segments with vessel diameters ≥1.5 mm were included for analysis. Reader 1 found 40 segments with stair-step artifacts, and reader 2 found 56 segments with stair-step artifacts. On a patient level, 22/50 (44%) (reader 1) and 23/50 (46%) (reader 2) scans showed stair-step artifacts in standard reconstructions, whereas with ZeeFree the occurrence of stair-step affected scans was significantly reduced to 8/50 (16%) (*P* < 0.001, respectively). The interrater agreement was good (α = 0.69).

There were no significant differences in image noise, SNR, and CNR between standard and ZeeFree reconstructions (*P* = 0.919, *P* = 0.958, *P* = 0.941, respectively) (Table 2).

**TABLE 2:** Objective Image Quality Metrics

Image Characteristics	Standard Reconstruction	ZeeFree Mode	*P*
Image noise, HU	59.6 ± 5.1	59.7 ± 5.3	0.919
SNR	8.0 ± 1.8	7.9 ± 1.8	0.958
CNR	9.6 ± 1.9	9.5 ± 1.9	0.941

Note: Data are mean ± standard deviation.

SNR, signal-to-noise ratio; CNR, contrast-to-noise ratio; HU, Hounsfield units.

The median scores for segments with stair-step artifacts in standard reconstructions were 2 and 3 for readers 1 and 2, respectively. Using the ZeeFree reconstructions, these segments had a median score of 1 for both readers. The improvement in scores was significant for both readers (each, *P* < 0.001).

Both readers consistently assigned all stair-step affected segments with an equal or a better score in ZeeFree compared with standard reconstructions. The application of the ZeeFree algorithm did not result in additional artifacts. For both readers, ZeeFree showed a significant reduction in stair-step artifact frequency from 40/504 affected segments (7.9%) in standard to 11/504 (2.2%) in ZeeFree reconstructions for reader 1, and from 56/504 (11.1%) affected segments in standard to 15/504 (3.0%) in ZeeFree reconstructions for reader 2 (*P* < 0.001, respectively).

In standard reconstructions, reader 1 considered 12/504 of all segments (2.4%) to be nondiagnostic (ie, score 4) due to stair-step artifacts, where with ZeeFree reconstructions this rate was significantly lower (3/504 segments, 0.6%, *P* < 0.05). Similarly, reader 2 found 11/504 segments (2.2%) of nondiagnostic image quality in standard reconstructions, whereas with ZeeFree only 3/504 segments (0.6%) remained nondiagnostic. This trend for reader 2 did not reach statistical significance (*P* = 0.056).

**FIGURE 3:**
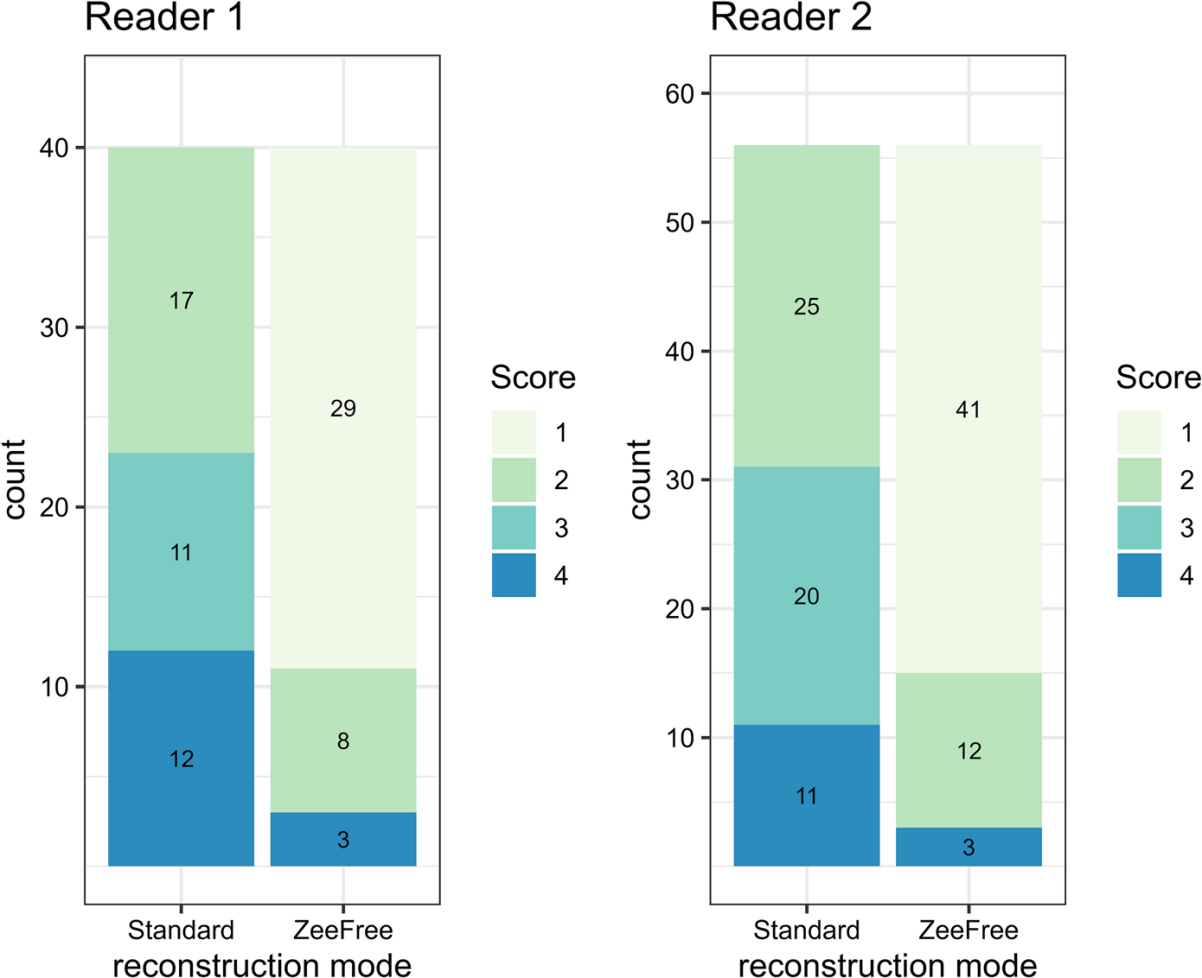
Stair-step artifact affected segments for standard and ZeeFree reconstructions.

**FIGURE 4:**
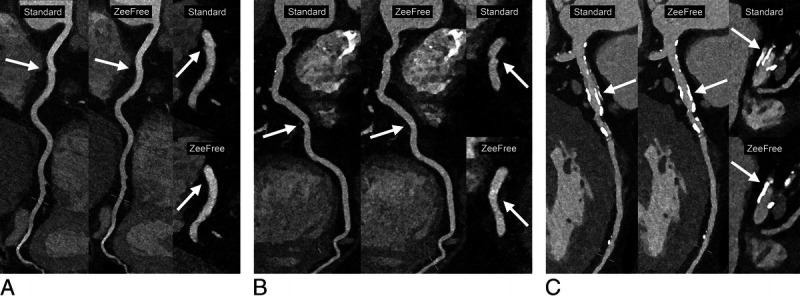
Two corresponding curved reformations of dual-source photon-counting detector CCTA in the standard (left) and in the ZeeFree mode (middle), and corresponding straight oblique reformations (right) in the same cardiac phase. A, A 45-year-old male patient (BMI, 28.5 kg/m^2^) with a mean heart rate of 65 bpm and a heart rate variability of 2.7 bpm (SD) and 6 bpm (range) showing stair-step artifacts in the RCA, which were resolved with the ZeeFree algorithm. B, A 64-year-old male patient (BMI, 32.2 kg/m^2^) with a mean heart rate of 60 bpm and a heart rate variability of 2.5 bpm (SD) and 7 bpm (range) showing stair-step artifacts in the RCA, which were resolved with the ZeeFree algorithm. C, A 77-year-old male patient (BMI, 24.9 kg/m^2^) with a mean heart rate of 81 bpm and a heart rate variability of 5.6 bpm (SD) and 13 bpm (range) showing stair-step artifacts in the LAD, which were resolved with the ZeeFree algorithm.

**FIGURE 5:**
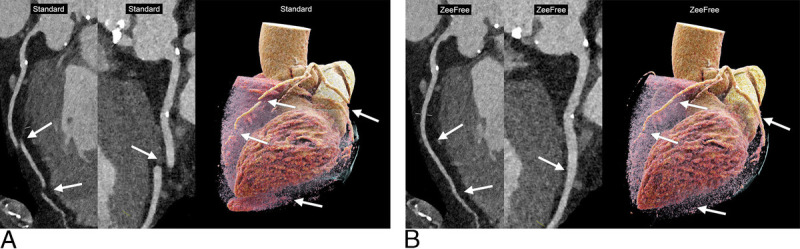
Curved planar reformations and cinematic rendering of dual-source photon-counting detector CCTA showing stair-step artifacts in the LAD, the CX, and at the left ventricular apex in a 75-year-old female patient (BMI, 30.1 kg/m^2^; mean heart rate, 75 bpm; heart rate variability, 3.4 bpm [SD] and 9 bpm [range], respectively). A, Standard reconstructions. B, ZeeFree reconstructions.

Figure 3 shows the distribution of segments with stair-step artifacts for both readers. Representative CCTA images with standard and with ZeeFree reconstructions are provided in Figures 4 and 5.

### Relationship of Stair-Step Artifacts With Heart Rate Characteristics

The occurrence of stair-step artifacts showed no significant relationship with the mean HR during data acquisition (*P* = 0.883 for reader 1 and *P* = 0.533 for reader 2). The frequency of nondiagnostic segments also was not significantly related to the mean HR (*P* = 0.872 for reader 1 and *P* = 0.800 for reader 2).

HRV_SD_ was significantly related to the occurrence of stair-step artifacts for reader 2 (*P* < 0.05), whereas for reader 1, the trend was not statistically significant (*P* = 0.077). For both readers, HRV_SD_ showed no significant relationship with the frequency of nondiagnostic segments (*P* = 0.662 for reader 1 and *P* = 0.881 for reader 2).

HRV_range_ showed a significant relationship with the occurrence of stair-step artifacts for reader 2 (*P* < 0.05) and only a trend for reader 1 (*P* = 0.080). The frequency of nondiagnostic segments was not significantly related to HRV_range_ for both readers (*P* = 0.549 for reader 1 and *P* = 0.753 for reader 2).

### Relationship of Stair-Step Artifacts With Patient Characteristics

Body mass index showed a significant relationship with the occurrence of step artifacts for reader 2 (*P* < 0.05) but not for reader 1 (*P* = 0.099). A high BMI was significantly related to the occurrence of nondiagnostic segments due to stair-step artifacts for both readers (*P* < 0.05, each).

Patient body weight showed no significant relationship with the occurrence of step artifacts for either reader, similar to patient height, age, and sex (all *P*'s > 0.05).

## DISCUSSION

Stair-step or misregistration artifacts are a well-known issue in sequential, prospectively ECG-triggered CCTA acquisitions.^4–6,22,23^ We introduced and evaluated a new algorithm to reduce stair-step artifacts in sequential CCTA using a nonrigid registration between the borders of 2 adjacent subvolumes. We found 8% (reader 1) and 11% (reader 2) of segments being affected by such artifacts in ultra-high-resolution CCTA with dual-source photon-counting detector CT. The occurrence of these artifacts showed a relationship to the HR variability and to the patients' BMI. Using a novel, so-called ZeeFree algorithm led to a significant reduction of stair-step artifact frequency and importantly, to a lower rate of segments with a nondiagnostic image quality.

Several previous studies have investigated stair-step artifacts in sequential CCTA acquisitions. Depending on the patient population studied, scanner type, vendor, and reconstruction phase, different frequencies for the occurrence of stair-step artifacts have been reported. Paul et al^4^ reported stair-step artifacts in 18% of scans in second-generation dual-source CT for best systolic reconstruction phases, whereas Jin et al^5^ reported an incidence of 78% with the same scanner. In our population, we found at least 1 segment affected by stair-step artifacts of variable severity in 44% (reader 1) and 46% (reader 2) of all scans.

Husmann et al,^6^ using a 64-slice single-source CT system, reported that stair-step artifacts occurring simultaneously in the chest wall and in coronary arteries were related to the patients' BMI and body weight. Stair-step artifacts of the coronary arteries alone did not show this relationship but were significantly related to HR variability. Muenzel et al,^7^ using a 256-slice single-source CT system, reported a significant relationship between stair-step artifacts and HR but did not find a significant relationship with HR variability. In our study using a dual-source photon-counting detector CT scanner, we found that stair-step artifacts in sequentially acquired ultra-high-resolution CCTA were significantly linked to HR variability and BMI. In addition, the prevalence of nondiagnostic segments due to stair-step artifacts was also related to the patients' BMI.

It was previously speculated that the relationship between stair-step artifacts and high BMI and/or high body weight may be caused by a relative movement between patient and CT table during rapid acceleration and deceleration inherent to sequential acquisitions. An additional explanation for this may be a limited breath-hold capability of obese people.^24^ High HR variability may lead to data acquisition in slightly different cardiac phases in sequential prospective ECG-triggering, which in turn may lead to misalignment or stair-step artifacts between adjacent subvolumes.^21,25^

With the aim to minimize the occurrence and extent of stair-step artifacts, the ZeeFree algorithm uses a nonrigid registration between the borders of 2 adjacent subvolumes. Using this algorithm, our investigation showed a considerable reduction in stair-step artifact occurrence by 73% for both readers (from 40 to 12 affected segments for reader 1 and from 56 to 15 affected segments for reader 2). Importantly, ZeeFree also reduced the occurrence of nondiagnostic segments because of stair-step artifacts by 75% (from 12 to 3 nondiagnostic segments) and 73% (from 11 to 3 nondiagnostic segments) for both readers, respectively. Beyond potentially improving the diagnostic accuracy of CCTA through the reduction of coronary segments with a nondiagnostic image quality, the algorithm may be also helpful to reduce the number of CCTA studies being not eligible for calculation of the fractional flow reserve (CT_FFR_) because of vessel misalignment.

There were, however, some stair-step artifacts that could not be corrected by the algorithm. Some of these uncorrected artifacts may arise when there is a gap between 2 adjacent subvolumes where no data are acquired. This gap may occur with patient or cardiac movement relative to the examination table along the *z*-axis, either due to gross patient movement, due to inertia of mass during acceleration and deceleration, mediastinum displacement secondary to an incomplete breath-hold, or physiologically throughout the cardiac cycle. The most critical combination is relative movement of the heart (by any of the means discussed above) along the *z*-axis in the opposite direction of the pitch movement between acquisitions of adjacent subvolumes, potentially causing a gap in the acquired data.^25^ The data missing between the respective subvolumes cannot be corrected or “generated” in postprocessing and thus may result in persistent, not correctable stair-step artifacts. Parallel movement to the pitch would result in a bigger overlap between the acquired subvolumes, also potentially causing stair-step artifacts by doubling of structures. However, in this case, no information would be lost in the acquisition process, and alignment of subvolumes may be corrected in postprocessing.^18^ Similarly, movement perpendicular to pitch direction would result in complete data acquisition with a lateral or anterior-posterior offset, which may also be corrected in postprocessing. Regarding breathing motion, expiration would cause a critical shift antiparallel to pitch movement by cranially displacing the heart, creating a gap of missing data.^26^ In contrast, inspiration would lead to a parallel shift relative to pitch movement, causing a “favorable” overlapping of the subvolumes.^8,25^

The following study limitations merit consideration. First, this study was conducted in a single center only. Second, the application of the algorithm evaluated in this study is limited to 1 vendor, and only a single type of CT scanner (ie, photon-counting detector CT^27–29^) was used, although the ZeeFree algorithm is compatible for other CT machines from the same vendor as well. Third, the number of subjects included was limited, which may be the reason why some statistical tests did not reach statistical significance. Fourth, we found a relatively high number of segments showing stair-step artifacts; however, this is related to the fact that a dedicated protocol with a narrow collimation was used. In addition, a large number of these artifacts did not result in nondiagnostic image quality. Finally, only subjective image quality was considered, and the effect of the algorithm on the diagnostic accuracy relative to the reference standard cardiac catheter angiography was not evaluated. However, when considering CCTA as a modality with an intent-to-diagnose purpose, nondiagnostic coronary segments must be counted as positive for disease.^10^ Thus, it can be expected that application of the algorithm would improve the specificity and positive predictive value of the test.

In conclusion, our study introduces and demonstrates the effectiveness of a new algorithm for significant reduction of stair-step artifact frequency and severity in sequential mode CCTA.
